# Hajdu-Cheney Syndrome as a Rare Cause of Acroosteolysis: A Case Report

**DOI:** 10.7759/cureus.112014

**Published:** 2026-07-03

**Authors:** Chaimae Nahi, Wafaa Jdioui, Khadija Mouaddine, Mohamed Amine Ichane, Nadia Mebrouk, Bouchra Chkirate

**Affiliations:** 1 Department of Pediatrics, Children’s Hospital of Rabat, Ibn Sina University Hospital Center, Faculty of Medicine and Pharmacy of Rabat, Mohammed V University, Rabat, MAR; 2 Department of Medical Genetics, Children’s Hospital of Rabat, Ibn Sina University Hospital Center, Faculty of Medicine and Pharmacy of Rabat, Mohammed V University, Rabat, MAR

**Keywords:** acroosteolysis, basilar invagination, bisphosphonates, case repor, craniofacial dysmorphism, hajdu–cheney syndrome, notch2, osteoporosis, skeletal dysplasia

## Abstract

Hajdu-Cheney syndrome (HCS) is a rare autosomal dominant multisystem disorder caused by mutations in the *NOTCH2* gene, leading to dysregulation of the Notch signaling pathway. It is characterized by a broad and variable clinical spectrum, including acroosteolysis, osteoporosis, short stature, craniofacial dysmorphism, and potential cardiovascular, renal, and neurological involvement.

We report the case of a 16-year-old boy, born to consanguineous parents, who was referred for evaluation of facial dysmorphism. Clinical examination revealed typical features of HCS, including brachycephaly with a prominent occiput, hypertelorism with telecanthus, low-set ears, long philtrum, micrognathia, thick hair and eyebrows, periodontal disease with early tooth loss, brachydactyly with pseudo-clubbing, and pectus carinatum. The patient had a history of surgically treated patent ductus arteriosus.

Radiological investigations showed kyphoscoliosis, generalized osteoporosis with multiple fractures, delayed closure of cranial sutures, an enlarged J-shaped sella turcica, and transverse acroosteolysis of the distal phalanges. Brain MRI revealed basilar invagination with compression of the acoustic-facial nerve bundle, explaining progressive bilateral hearing loss. Laboratory findings were within normal limits, with no abnormalities in calcium-phosphate metabolism or inflammatory markers.

Whole-exome sequencing identified a heterozygous nonsense mutation in the *NOTCH2* gene (NM_024408.4.6484del), classified as likely pathogenic, confirming the diagnosis of HCS. The patient was treated with IV zoledronic acid every six months, along with calcium and vitamin D supplementation.

This case illustrates the multisystem and highly variable presentation of HCS. The combination of characteristic skeletal, craniofacial, neurological, and cardiovascular features associated with a de novo *NOTCH2* mutation broadens the clinical spectrum of this exceptionally rare disorder. Advances in understanding the role of Notch signaling may lead to targeted therapies in the future. Prognosis depends on the severity of systemic complications, particularly neurological involvement and thoracic deformities leading to respiratory impairment.

## Introduction

Hajdu-Cheney syndrome (HCS) is a rare multisystem disorder inherited in an autosomal dominant manner [1]. It is characterised by acroosteolysis, a pathological process involving progressive osteolysis of the terminal phalanges and considered one of the cardinal features of the syndrome, osteoporosis, short stature, distinctive craniofacial features, as well as neurological, cardiovascular, and renal abnormalities, including polycystic kidneys [1]. HCS belongs to the broader group of osteolysis syndromes [2]. It is referenced in the Online Mendelian Inheritance in Man (OMIM) database under the number 102500 and in Orphanet as ORPHA955. The syndrome is also known by several alternative names, including acro-dento-osteodysplasia, acroosteolysis with osteoporosis and cranio-mandibular abnormalities, arthrodentoosteodysplasia, and serpentine fibula-polycystic kidney syndrome.

Clinically, HCS exhibits a wide and variable spectrum, which is rarely fully expressed in a single individual. Reported cases demonstrate heterogeneous manifestations that tend to progress with age, evolving from early childhood through adulthood [2]. The condition was first described by Hajdu N and Kauntze R in 1948 [3] and later by Cheney WD in 1965 in a familial context, supporting its autosomal dominant mode of inheritance [4].

HCS is an extremely rare condition, with a prevalence estimated at less than 1 per 1,000,000 individuals [5]. It is caused by heterozygous mutations in the *NOTCH2* gene [5], located on chromosome 1p13-p11. Although most cases follow an autosomal dominant inheritance pattern, sporadic cases have also been reported [5].

Given the exceptional rarity of HCS and its marked clinical heterogeneity, documenting additional cases remains essential to broaden the current understanding of its phenotypic spectrum, natural history, and diagnostic challenges. Furthermore, the limited number of cases reported in the literature continues to hinder the development of evidence-based management strategies. Therefore, each new observation contributes valuable information that may improve recognition of the disease and enhance patient care.

This clinical case was reported in accordance with the Surgical CAse REport (SCARE) criteria [6].

## Case presentation

This is a 16-year-old boy, born to first-degree consanguineous parents, with no family history of acroosteolysis and healthy siblings, who underwent surgery at the age of 8 for patent ductus arteriosus and was referred to the Rabat Paediatric University Hospital for facial dysmorphism. The patient attended our department in October 2025 and presented with brachycephaly with a prominent occiput, hypertelorism with telecanthus, low-set ears, a long philtrum, and micrognathia. The eyebrows and eyelashes were thick, and the hair was straight with a high hairline. He also presented with periodontal disease with early and progressive tooth loss, brachydactyly with a pseudo-Hippocratic digital appearance, and pectus carinatum (Figure 1).

**Figure 1:**
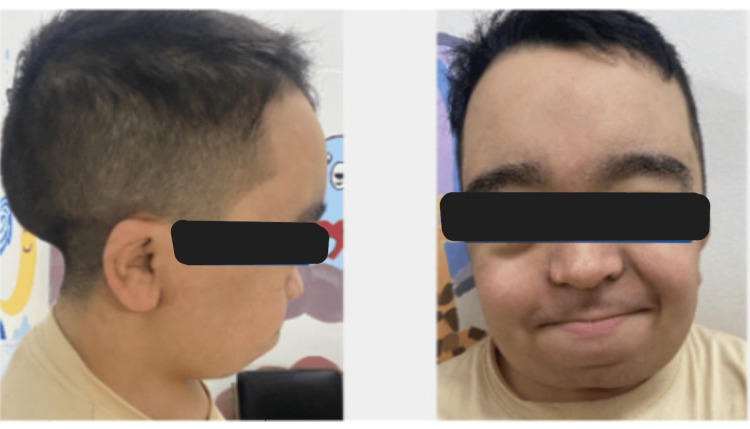
Clinical image of the patient showing brachycephaly characterized by a prominent occiput, hypertelorism with telecanthus, low-set ears, a long philtrum, and micrognathia. The patient also has thick eyebrows and eyelashes, and straight hair with a high hairline.

For further evaluation, X-rays and laboratory tests were carried out. Radiographically, standard X-rays of the spine showed mild kyphoscoliosis and signs of osteoporosis in the form of a reduction in vertebral body height (Figure 2). Our patient had multiple fractures of varying ages and diffuse demineralisation of the bone matrix throughout the body. Regarding the skull, standard X-rays showed delayed closure of the sutures, an enlarged J-shaped sella turcica, and dental malalignment. X-rays of the hands showed osteolytic transverse bands of the distal phalanges consistent with transverse acro-osteolysis (Figure 3). Bone age was estimated to be between 10 and 11 years.

**Figure 2:**
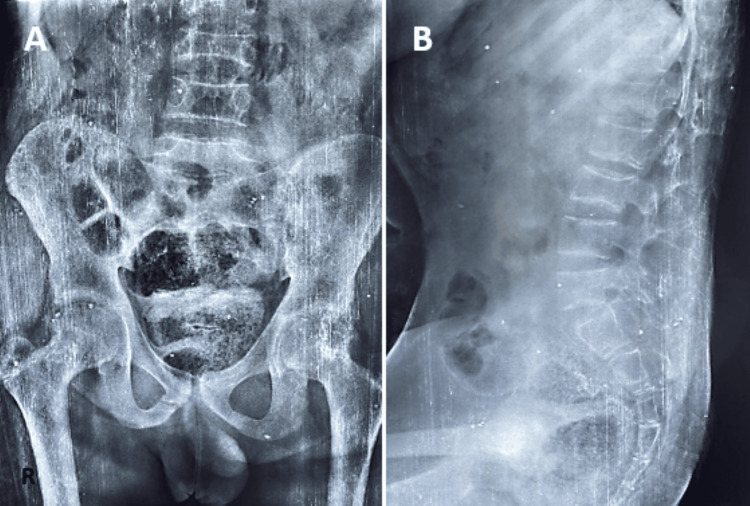
Standard X-ray images of the spine showing mild kyphoscoliosis and signs of osteoporosis. A: Standard anteroposterior X-ray of the pelvis and lumbar spine showing diffuse demineralisation. B: Standard lateral X-ray of the lumbosacral spine showing mild kyphoscoliosis and reduced vertebral body height.

**Figure 3:**
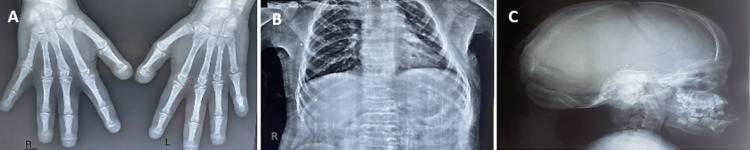
X-ray image showing diffuse demineralisation of the bone matrix throughout the body. A: X-ray of the hands showing osteolytic transverse bands in the distal phalanges, consistent with transverse acroosteolysis. B: X-ray of the rib cage showing diffuse bone demineralisation of the ribs, with thinning of the costal arches and cortical layer. C: Standard skull X-ray showing delayed suture closure, an enlarged J-shaped sella turcica, and dental malalignment.

Brain MRI demonstrated basilar impression with compression of the right acoustic-facial nerve bundle and hypertrophy of the cranial vault. The abnormal osseous changes exhibited low signal intensity on T1-weighted images and heterogeneous intermediate-to-high signal intensity on T2-weighted sequences, without evidence of intracranial mass effect, findings consistent with chronic osseous remodeling associated with the syndrome. These abnormalities were correlated with progressive bilateral hearing loss (Figure 4).

**Figure 4:**
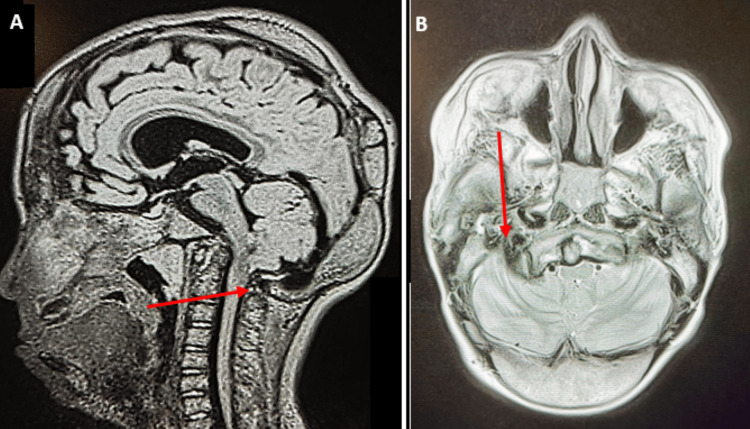
Brain MRI showing basilar impression with compression of the right acoustic-facial nerve bundle and a hypertrophied appearance of the cranial vault, associated with progressive bilateral hearing loss. A: Sagittal brain MRI section showing basilar impression; the arrow indicates upward displacement of the odontoid process. B: Axial brain MRI image showing compression of the right acoustic-facial nerve bundle indicated by the arrow.

Lumbosacral CT revealed a comminuted fracture involving the right side of the S1 vertebral body. The fracture was associated with diffuse osteopenia, manifested by generalized decreased bone density and cortical thinning. Areas of marginal sclerosis (hyperdense condensation) surrounded the fracture site, suggesting chronic remodeling, while the remaining sacral bone displayed heterogeneous hypodensity related to severe demineralisation (Figure 5).

**Figure 5:**
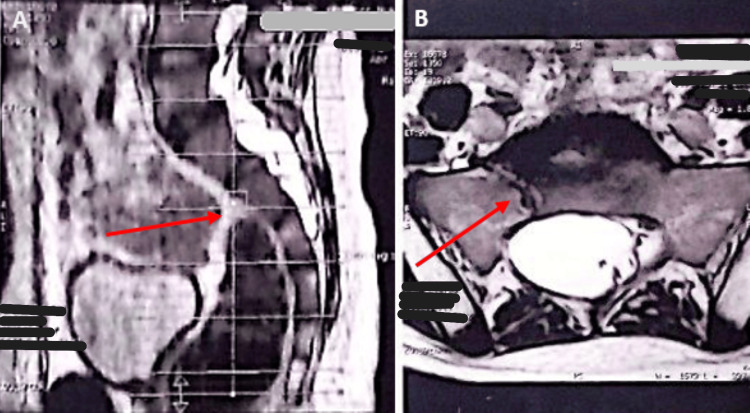
Lumbosacral computed tomography showing a comminuted fracture of the right side of the S1 vertebral body, with marginal sclerosis and diffuse bone demineralisation. A: Sagittal section from a lumbar CT scan; the arrow indicates the biconcave appearance of the lumbar vertebral bodies. B: Axial CT image; the arrow indicates a comminuted fracture of the right side of the S1 vertebral body.

A bone densitometry scan using dual-energy X-ray absorptiometry (DXA) was performed, and the results showed a spinal Z-score of -4.5 (Figure 6). Laboratory results were entirely normal. There were no abnormalities in the calcium-phosphorus profile or evidence of a biological inflammatory syndrome.

**Figure 6:**
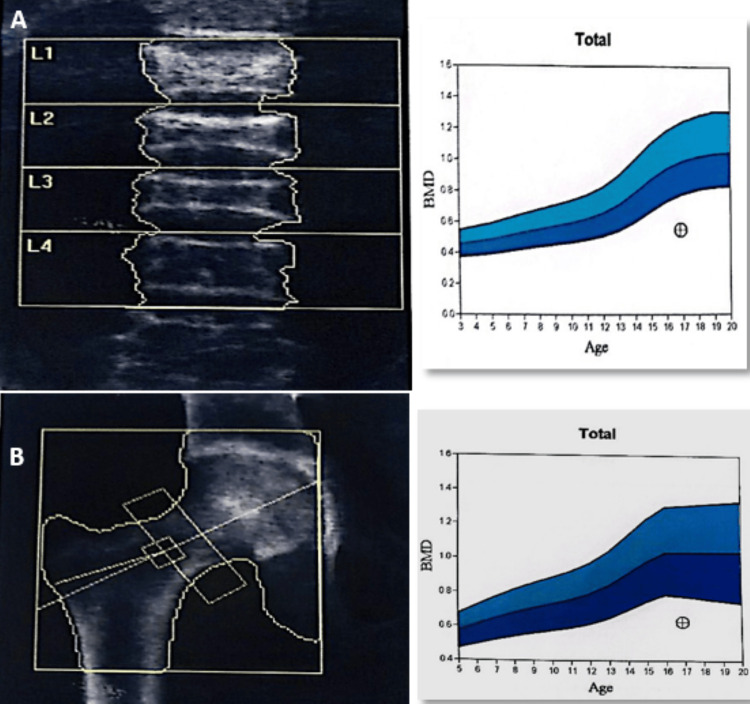
Bone densitometry scan using dual-energy X-ray absorptiometry (DXA). A: Lumbar spine bone densitometry (116×110) scan showing osteoporosis, with a Z-score of -4.5. B: Right hip bone densitometry (85×100, neck 48×15, hal 91 mm) scan showing osteoporosis, with a Z-score of -3.2.

Molecular analysis via whole-exome sequencing identified a heterozygous mutation in the *NOTCH2* gene (variant NM_024408.4.6484del). Given that the patient’s parents are healthy, this was considered likely to be a de novo mutation in this family.

Following the diagnosis of HCS in December 2025, treatment was initiated with IV zoledronic acid at a dose of 0.05 mg/kg every six months, together with calcium and vitamin D supplementation. Baseline laboratory investigations, including serum calcium, phosphate, and renal function tests, were within normal limits.

The patient was followed for a period of eight months. During follow-up, treatment was well tolerated both clinically and biologically, with no infusion-related adverse events, hypocalcaemia, renal impairment, or other treatment-related complications.

Clinical evolution was favourable, with a significant reduction in bone pain and no occurrence of new pathological fractures. Follow-up bone densitometry demonstrated an improvement in bone mineral density, with the lumbar spine Z-score increasing from -4.5 at baseline to -3.8 after eight months of treatment.

Given the presence of basilar impression at diagnosis, regular neurological and radiological monitoring was performed. Follow-up imaging showed morphological stability of the basilar impression, without evidence of progression or new neurological complications. Bilateral hearing loss remained stable throughout the follow-up period.

Owing to the progressive and multisystem nature of HCS, long-term multidisciplinary follow-up has been maintained. The patient continues to receive periodic clinical, radiological, and densitometric assessments to monitor disease progression and response to therapy.

## Discussion

Before the molecular basis of HCS was identified, Brennan AM and Pauli RM outlined ten principal clinical features of the disorder, namely acroosteolysis, the presence of Wormian bones, platybasia, early tooth loss, micrognathia, coarse facial characteristics, thick hair, midfacial flattening, short stature below the 5th percentile, and a positive family history [1]. They also introduced a diagnostic framework integrating clinical criteria with inheritance patterns [1].

The wide clinical variability observed in HCS is largely attributed to mutations in the *NOTCH2* gene, as the Notch signalling pathway plays a crucial role in embryonic development [7]. Disruptions in this pathway may result in multisystem developmental abnormalities involving organs such as the skeleton, heart, liver, kidneys, eyes, face, and vascular system. Molecular analyses have identified numerous nucleotide variants and protein alterations linked to HCS, which likely account for the broad phenotypic heterogeneity reported in the literature.

Clinically, craniofacial dysmorphism typically emerges during infancy or early childhood, initially being subtle but progressively becoming more evident. Characteristic features include brachycephaly with a prominent occiput [8], mild hypertelorism with telecanthus, downward and outward slanting palpebral fissures, thick eyebrows sometimes meeting in the midline (synophrys), low-set ears, an elongated philtrum, micrognathia with a high-arched or cleft palate, and a short neck [8]. Hair, eyebrows, and eyelashes are often dense, with straight hair and a high hairline [8].

Cranial skeletal abnormalities are almost constant and include frontal sinus hypoplasia, Wormian bones, and occasionally dolichocephaly [8]. Basilar invagination and platybasia are common and may disrupt CSF dynamics, potentially leading to an enlarged empty sella turcica or syringomyelia [9]. These anomalies can also give rise to neurological complications, particularly in adolescence and adulthood, due to cranial nerve stretching and cervical spine instability, sometimes associated with Arnold-Chiari malformation [9].

Defective ossification of long bones explains the short stature typically observed, usually ranging from 140 to 155 cm. A frequently reported skeletal feature is a serpentine fibula, which may be associated with polycystic kidney disease as part of serpentine fibula-polycystic kidney syndrome (SFPKS) [10]. Increasing evidence suggests that HCS and SFPKS may represent variable expressions of a single disease entity [11,12].

Primary acroosteolysis is a common finding, usually bilateral and symmetrical, and tends to appear later in childhood. It is often painless but can occasionally be associated with inflammatory pain [8] and swelling of the distal extremities, mimicking digital clubbing. This process involves progressive resorption of the distal phalanges, leading to shortening of the digits [13]. In HCS, osteolysis typically has a transverse, band-like pattern affecting the mid-portion of the distal phalanges while sparing the proximal and distal ends. It may also involve the metacarpals, metatarsals, and forearm bones [11].

Generalised osteoporosis is frequently reported [14,15], predisposing patients to multiple vertebral fractures and secondary deformities such as biconcave vertebrae, scoliosis, and spondylolisthesis. Despite these skeletal abnormalities, calcium and phosphate metabolism remain normal. Experimental work by Canalis E et al., using a mouse model with an activating *NOTCH2* mutation, demonstrated increased activity of both osteoclasts and osteoblasts, resulting in accelerated bone turnover and enhanced bone resorption in both cortical and trabecular compartments. This effect was not observed in NOTCH1 mutations [15].

Joint and spinal hyperlaxity are also common [8], often leading to postural abnormalities such as kyphosis, scoliosis, or spondylolisthesis, frequently associated with pain. In some cases, acroosteolysis may precede joint manifestations. Cranial abnormalities may contribute to headaches and cranial nerve dysfunction, including hearing impairment and oculomotor disturbances [9]. Thoracic deformities, related to skeletal dysplasia and ligamentous laxity, may result in chronic respiratory insufficiency and recurrent infections [8].

Cardiac anomalies have been described, including congenital defects such as aortic coarctation, ventricular septal defects, patent ductus arteriosus, and progressive myxomatous degeneration of the aortic valve [8]. Although polycystic kidney disease is uncommon, it represents a key feature in a subset of patients, particularly within the SFPKS spectrum [7-10].

Additional features may include dermatological manifestations such as hirsutism, seborrhea, and palmoplantar hyperhidrosis [8,16]. Pituitary insufficiency may occur secondary to cranial abnormalities. Other reported anomalies include early umbilical hernia and dental malocclusion. Hearing loss may be conductive, due to bone abnormalities, or sensorineural, resulting from nerve compression. Periodontal disease with early and progressive tooth loss is frequently observed [8].

In the present case, the patient exhibited typical craniofacial features of HCS, along with short stature, scoliosis, patent ductus arteriosus, and hearing impairment.

Laboratory investigations are generally unremarkable, with normal calcium and phosphate levels and no evidence of systemic inflammation, as also observed in our patient. Radiographic findings may include acroosteolysis, transverse osteolysis, serpentine fibula, craniofacial abnormalities, and spinal deformities or fractures. Additional findings may include skull flattening, an elongated sella turcica, and reduced vertebral bone density, detectable through imaging and bone mineral density (BMD) assessment [17].

These clinical and radiological features are not specific and may overlap with other conditions such as osteogenesis imperfecta or mucopolysaccharidoses, making whole-exome sequencing essential for definitive diagnosis.

At the molecular level, HCS is associated with nonsense mutations or deletions in exon 34 of the *NOTCH2* gene, leading to a premature stop codon upstream of the proline, glutamic acid, serine, and threonine (PEST) domain [17]. In our case, a heterozygous nonsense mutation (NM_024408.4.6484del) was identified, which has been reported as likely pathogenic in the literature [5].

Currently, no curative treatment exists for HCS. Management is primarily supportive and aims to counteract the increased bone resorption resulting from enhanced osteoclast-mediated activity [18]. Bisphosphonates are the most commonly used therapeutic agents in this context [19], owing to their strong affinity for bone tissue and their inhibitory effect on osteoclast-mediated resorption [20].

Mutations affecting the terminal exon of *NOTCH2* result in persistent activation of the Notch signalling pathway due to impaired degradation of the receptor. This suggests that *NOTCH2* could represent a promising therapeutic target in the future, potentially allowing for disease-modifying interventions rather than purely symptomatic treatment. Experimental strategies aimed at modulating Notch signalling, such as monoclonal antibodies targeting the receptor or its ligands, or peptides that disrupt the transcriptional complex, are currently under investigation [21].

The prognosis of HCS is influenced by the severity of complications, particularly neurological involvement due to basilar invagination and respiratory compromise resulting from thoracic deformities. Given the rarity of the condition and the limited data on patient-reported outcomes, assessing long-term quality of life and life expectancy remains challenging.

## Conclusions

This case describes a 16-year-old patient with HCS caused by a heterozygous de novo *NOTCH2* mutation, presenting with a distinctive combination of craniofacial dysmorphism, transverse acroosteolysis, severe osteoporosis with multiple fractures, patent ductus arteriosus, basilar impression, and progressive hearing loss. This observation highlights the considerable phenotypic variability of the syndrome and illustrates how skeletal, neurological, and cardiovascular manifestations may coexist in the same patient. Despite extensive bone involvement, biochemical investigations remained normal, emphasizing the dissociation between laboratory findings and disease severity. This report expands the clinical spectrum associated with HCS and underscores the value of integrating clinical, radiological, and molecular findings to characterize this rare disorder.
